# Oxygen desaturation during the 6-min walk test as a risk for osteoporosis in non-cystic fibrosis bronchiectasis

**DOI:** 10.1186/s12890-019-0794-x

**Published:** 2019-02-04

**Authors:** Hung-Yu Huang, Te-Fang Sheng, Chang-Wei Lin, Ting-Wen Wang, Chun-Yu Lo, Fu-Tsai Chung, Lan-Yan Yang, Yu-Bin Pan, Chun-Hua Wang

**Affiliations:** 1 Division of Pulmonary and Critical Care, Department of Internal Medicine, Saint Paul’s Hospital, Taoyuan, Taiwan; 2 Department of Thoracic Medicine, Chang Gung Memorial Hospital and College of Medicine, Chang Gung University, 199 Tun-Hwa North Road, Taipei, 105 Taiwan; 3 College of Medicine, Chang Gung University, Taoyuan, Taiwan; 4 Department of Physical Medicine and Rehabilitation, National Taiwan University Hospital, Taipei, Taiwan; 5 Biostatistics Unit, Clinical Trial Center, Chang Gung Memorial Hospital, Taoyuan, Taiwan

**Keywords:** Desaturation, Non-cystic fibrosis bronchiectasis, Bone mineral density, 6-min walk test, High-resolution computed tomography

## Abstract

**Background:**

Osteoporosis is a common comorbidity in non-cystic fibrosis (non-CF) bronchiectasis patients. We determined whether desaturation during 6-min walk test (6MWT) can be a predictor for osteoporosis risk.

**Methods:**

This was a retrospective cross-sectional study. Sixty-six non-CF bronchiectasis patients were enrolled. Lung function, walking distance, the lowest oxygen saturation (SpO_2_), the fall in SpO_2_ (ΔSpO_2_), and the distance–saturation product (DSP) were determined during the 6MWT. Desaturators (*n* = 45) were defined as those with ΔSpO_2_ > 10% or the lowest SpO_2_ < 88%. Bone mineral density (BMD) was determined through dual-energy X-ray absorptiometry. The severity of non-CF bronchiectasis was evaluated using high-resolution computed tomography.

**Results:**

Osteoporosis was evident in more desaturators (82%) than non-desaturators (43%, *p* < 0.01). BMD at the level of the femoral neck was significantly lower in desaturators than in non-desaturators (− 3.6 ± 1.1 vs. − 2.4 ± 0.9, *p* < 0.01). BMD was correlated positively with the lowest SpO_2_ and negatively with ΔSpO_2_ and severe exacerbations. In multivariate linear regression analysis, desaturation during 6MWT was the most significant predictive factor for osteoporosis (95% confidence interval − 1.60 to − 0.26, *p* = 0.01). Other risk factors included old age, low body mass index and severe exacerbation.

**Conclusions:**

Exertional desaturation during the 6MWT was a significant predictive factor for osteoporosis in Asian non-CF bronchiectasis patients. The 6MWT may be useful in identifying the osteoporotic phenotype of non-CF bronchiectasis and increasing clinician awareness to promote early intervention.

## Background

Non-cystic fibrosis (non-CF) bronchiectasis is a chronic lung disease characterized by irreversibly dilated and damaged bronchi and bronchioles [1]. It is associated with several important clinical traits, namely, poor mucus clearance, airflow obstruction, and chronic systemic inflammation. With disease progression, there is deterioration of pulmonary function, diminution of exercise capacity, and an increase in mortality [2, 3]. Comorbidities such as gastroesophageal reflux disease, pulmonary hypertension, and osteoporosis frequently occur with non-CF bronchiectasis [3, 4].

Osteoporosis is one of the most common comorbidities of non-CF bronchiectasis [3]. The prevalence of diminished bone density in patients with non-CF bronchiectasis is high with more than 25% of the patients having osteoporosis and approximately 40–80% having osteopenia [5]. Risk factors for osteoporosis include age, gender, nutrition, inflammation, and ethnicity [6]. In other chronic respiratory diseases such as cystic fibrosis, chronic obstructive pulmonary disease (COPD) and obstructive sleep apnea, bone mineral density (BMD) scores have been associated with pulmonary function, hypoxia and systemic inflammation [7–10]. However, these risk factors associated with osteoporosis have not been completely explored in patients with non-CF bronchiectasis.

The six-minute walk test (6MWT) is a simple test to assess lung function and oxygen saturation during walking, which has been widely used in the clinical follow-up of patients with cardiopulmonary diseases, such as COPD, bronchiectasis, and idiopathic pulmonary fibrosis [11–14]. Desaturation during 6MWT has been used to predict the risk of dying from bronchiectasis [13]. Hypoxia may affect bone density through hypoxia-inducible factor (HIF) accumulation [15], which stimulates osteoclast activities [16], directly inhibits mesenchymal stem cells osteogenic differentiation [17], and attenuates the parathyroid hormone anabolic actions on bone formation in mature bone [18]. Hypoxia increases bone damage induced by acidosis and inflammation. The episodic hypoxia during daily activities seen in non-CF bronchiectasis may cause recurrent ischemic injury, which induces inflammation and an acidotic microenvironment in bone. In addition, hypoxia plays a role in NLRP3 (nucleotide-binding domain, leucine-rich-containing family, pyrin domain-containing-3 or Nod-like receptor protein 3) inflammasome activation [19], during bacterial infection with *Pseudomonas aeruginosa*, thus leading to the release of interleukin (IL)-1β with the induction of pulmonary inflammation [20]. Airway secretions of non-CF bronchiectasis patients with *Pseudomonas* or other chronic infection contain higher levels of IL-1β [20] which is a powerful proinflammatory cytokine stimulating osteoclastogenesis and increasing in vitro and in vivo bone loss [21]. Because exertional desaturation is a common manifestation after chronic lung destruction in non-CF bronchiectasis [13], we hypothesize that bronchiectasis patients who desaturated during exercise may develop osteoporosis demonstrated by a reduction in bone mineral density. There is scarcity of data concerning the contribution of exertional desaturation to the risk of developing osteoporosis in non-CF bronchiectasis. Therefore, we investigated the associated risk factors for osteoporosis based on the presence or absence of desaturation during 6-min walk test in Asian non-CF bronchiectasis patients.

## Methods

### Study population

Sixty-six non-CF bronchiectasis subjects were recruited from our outpatient clinic at Chang Gung Memorial Hospital between 2009 and 2017. Diagnosis of bronchiectasis was mostly based on HRCT. In only four patients, diagnosis was made by chest radiography and clinical history. These patients declined to have an HRCT performed, but the evidence for bronchiectasis was clear on chest radiography. As bone mineral density *(*BMD*)* test and 6-min walk test were not routinely performed for non-CF bronchiectasis, 66 subjects were our maximum evaluable cases and were not a convenience sample for this study. We used the femoral neck bone density T-score to calculate the power of the study. Based on this sample size of 21 non-desaturators and 45 desaturators, we achieved 94% power to detect a difference using mean (SD) BMD of − 2.4(1.2) and − 3.6(1.5) for non-desaturators and desaturators, respectively, based on the two-sample t test for equality at significance level of 5%. The inclusion criteria were as follows: non-CF bronchiectasis in a steady state defined as no changes in respiratory symptoms over the past 3 weeks and absence of other major pulmonary diagnoses. All women were post-menopausal. Patients were excluded if etiology of bronchiectasis was primary ciliary dyskinesia, common variable immunodeficiency, allergic bronchopulmonary aspergillosis, use of antibiotics within last one month, comorbid with hepatic failure or malignancy. All subjects completed 6MWT and dual-energy X-ray absorptiometry (DXA). The Chang Gung Memorial Hospital Institutional Review Board approved the study (IRB number: 201701886B0).

Desaturation during the 6MWT was defined as ΔSpO_2_ more than 10% compared with baseline SpO_2_ or lowest SpO_2_ during 6MWT less than 88% [11]. Medical records were inspected to calculate the incidence of emergency room (ER) visits and hospitalizations. Severe exacerbation was defined as ER visits or hospitalizations rate with a primary discharge diagnosis of bronchiectasis-related exacerbations [22]. Medical records revealed that 16 non-CF bronchiectasis patients had short-term systemic corticosteroid treatment for acute exacerbation during hospitalizations.

### BMD *assessment*

BMD was measured by DXA (Hologic Inc., Bedford, MA, USA) at femoral neck and lumbar spine (L2-L4). BMD units are in gram per square centimeter and expressed as T-score. The T-score is defined as the number of standard deviations above or below the mean BMD of a gender-matched normal population aged 26–30 years with peak bone mass. Osteoporosis was defined as a T- score ≤ 2.5 SD. Osteopenia was defined as a T-score between − 1 and − 2.5 SD according to WHO criteria [23]. The lowest T-score of femoral neck, lumbar spine and proximal hip is commonly recommended to be used for diagnosis of osteoporosis [24].

### HRCT score

Bronchiectatic changes of each lobe of both lungs were scored on a scale of 0 to 3 (0 = no bronchiectasis, 1 = one bronchopulmonary segment involved, 2 = more than one bronchopulmonary segment involved, and 3 = gross cystic bronchiectasis). Left lingula served as a separate lobe and the maximum score of the total 6 lobes was 18 points [25].

### 6 min walking test

All participants performed the 6MWT according to the recommendations of the ATS guideline [26]. The participants were instructed to walk back and forth in a 35-m corridor for 6 min. Exertional oxygen saturation (SpO_2_) was measured during the walking period by pulse oximetry (Criticare Systems Inc., Waukesha, WI, US). 6 min walking distance (6MWD) was determined after the participant stopped walking. Pulmonary function test including forced expiratory volume in one second (FEV_1_), forced vital capacity (FVC), and FEV_1_/FVC ratio, was performed before and after the 6MWT with spirometer (ST-250, Fukuda Sangyo Co. Ltd., Chiba, Japan). The distance–saturation product (DSP) was defined as the product of 6MWD (meter) and the lowest SpO_2_ (%) during 6MWT [13].

### Statistical analysis

We reported means, standard deviations (SD), and 95% confidence interval (95% CI) for the continuous variables and frequency or percentage for the categorical variables. Continuous variables were compared between two groups using t-test or Mann-Whitney test for even or uneven distribution, and categorical variables were compared using Chi-square test or Fisher’s exact test, as appropriate. We used Kolmogorov–Smirnov test to analyze the normality and homogeneity. Pearson correlation coefficient was used to ascertain the linear relationships between BMD and the variables. Univariate and multivariate linear regression analysis were used to estimate the linear relationship between the variables and femoral neck BMD, and the independent factors of the linear model were identified through a stepwise process. For the multivariate analysis, we initially entered all the variables in univariate analysis to fit the model, and the independent factors for BMD were identified under stepwise regression analysis to address the co-linear issue between variables. Statistical analyses were performed using SAS version 9.2 (SAS Institute, Cary, North Carolina, USA). A *p*-value < 0.05 was considered statistically significant.

## Results

Among the 66 non-CF bronchiectasis patients, 45(68%) were desaturators and 21(32%) were non-desaturators (Table 1). There was no significant difference in age, gender, or body mass index distribution between the groups. For all included patients, the prevalence of osteoporosis was 70% and that of osteopenia was 23%. The proportion of desaturators with osteoporosis (37/45, 82%) was higher than that of non-desaturators (9/21, 43%, *p* < 0.01).

**Table 1:** Clinical characteristics of patients with non-CF bronchiectasis

	Total (*N* = 66)	Non-desaturators (*N* = 21)	Desaturators (*N* = 45)	P
Age, yrs	65.2 ± 10.2 (62.6~67.7)	65.2 ± 8.6 (61.2~69.1)	65.2 ± 11.0(61.8~68.5)	0.930
Gender, female	51	14	37	0.210
HRCT score	10.5 ± 4.4 (9.4~11.7)	7.7 ± 3.6 (6.0~9.3)	11.9 ± 4 (10.7~13.2)	< 0.001
BMI (kg/m^2^)	21.8 ± 4.1 (20.8~22.8)	22.1 ± 2.9 (20.8~23.4)	21.7 ± 4.6 (20.3~23.1)	0.662
BMD T score				
Femoral neck	−3.2 ± 1.2 (−3.5~ − 2.9)	−2.4 ± 0.9 (− 2.7~ − 2.0)	− 3.6 ± 1.1 (−3.3~ − 3.9)	< 0.001
Lumbar spine	−2.3 ± 1.2 (− 2.6~ − 2.0)	−1.6 ± 0.9 (− 2.0~ − 1.1)	−2.6 ± 1.2 (− 3.0~ − 2.3)	0.001
Proximal hip	− 2.5 ± 1.2 (− 2.8~ − 2.2)	− 1.5 ± 0.8 (− 1.9~ − 1.2)	− 2.9 ± 1.0 (− 3.2~ − 2.6)	< 0.001
Osteoporosis, %	46 (70%)	9 (43%)	37 (82%)	0.005
Osteopenia, %	15 (23%)	9 (43%)	6 (13%)	
Pulmonary function				
FVC, L	1.7 ± 0.7 (1.6~1.9)	2.2 ± 0.7 (1.9~2.5)	1.5 ± 0.6 (1.3~1.7)	< 0.001
FVC, %	71.3 ± 21.7 (66.0~76.6)	81.4 ± 20.1 (72.2~90.5)	66.6 ± 21 (60.3~72.9)	0.009
FEV_1_, L	1.2 ± 0.5 (1.1~1.3)	1.6 ± 0.5 (1.3~1.8)	1.0 ± 0.5 (0.9~1.2)	< 0.001
FEV_1_, %	62.5 ± 24.0 (56.6~68.4)	71.8 ± 21.2 (62.2~81.5)	58.1 ± 24.2 (50.9~65.4)	0.024
FEV_1_/FVC, %	69.7 ± 10.9 (67.1~72.4)	71.2 ± 9.7 (66.8~75.7)	69 ± 11.4 (65.6~72.5)	0.449
6MWT parameters				
SpO_2_ saturation				
Pre-exercise,%	93.6 ± 4.1 (92.6~94.6)	95.0 ± 2.0 (94.1~96.0)	92.9 ± 4.6 (91.5~94.3)	0.197
Post-exercise,%	84.1 ± 7.8 (82.2~86.0)	92.1 ± 2.2 (91.1~93.1)	80.4 ± 6.5 (78.5~82.4)	< 0.001
Borg score				
Pre-exercise	1.0 ± 1.3 (0.7~1.4)	0.5 ± 1.0 (0.1~1.0)	1.3 ± 1.4 (0.8~1.7)	0.032
Post-exercise	4.4 ± 1.5 (4.0~4.7)	3.4 ± 0.9 (2.9~3.8)	4.9 ± 1.4 (4.5~5.3)	< 0.001
6MWD, M	417.7 ± 119.8 (388.2~477.1)	487.0 ± 65.0 (457.4~516.6)	385.3 ± 126.1 (347.4~423.2)	< 0.001
Severe exacerbation (times/year)	2.3 ± 4.0 (1.3~3.3)	0.8 ± 1.3 (0.2~1.4)	3.0 ± 4.6 (1.6~4.4)	0.023
Short-term systemic corticosteroid loading^a^ (days)	2.9 ± 6.6 (1.3~4.5)	0.7 ± 2.1 (− 0.3~1.6)	4.0 ± 7.7 (1.7~6.3)	0.0496

Note: Data are presented as mean ± standard deviation, or number (percentage)

Abbreviations: *CF* cystic fibrosis, *HRCT* high resolution computed tomography, *BMI* body mass index, *BMD* bone mineral density, *FEV*_*1*_ forced expiratory volume in 1 s, *FVC* forced volume capacity, *6MWT* six minute walk test, *SpO*_*2*_ oxygen saturation by pulse oximetry, *6MWD* six minute walk distance

The data in the parenthesis of the continue variables indicate 95% confidence interval (CI)

^a^Short-term systemic corticosteroid dosing: total days of prednisolone 20 mg per person

The desaturators during 6MWT were characterized by a significantly lower FEV_1_, FVC and 6MWD, higher HRCT scores and severe exacerbations compared to non-desaturators (Table 1). The BMD at the levels of femoral neck, lumbar spine and proximal hip in the desaturators were all significantly lower than those of non-desaturators (Table 1).

**Fig. 1:**
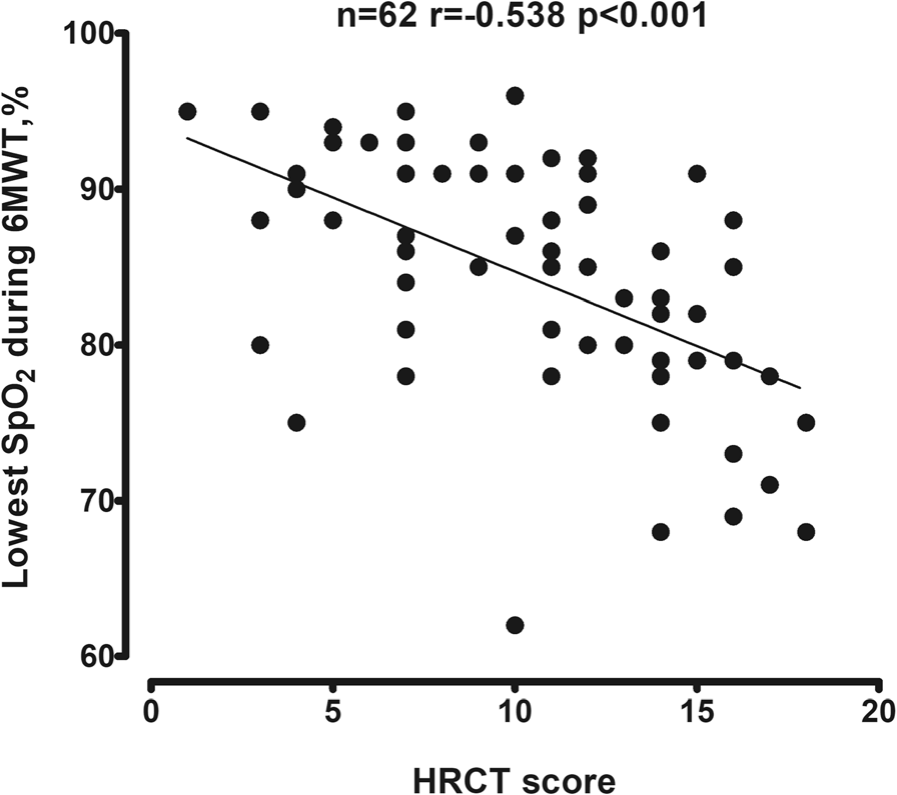
Correlation between HRCT score and the lowest SpO_2_ during 6MWT. The number and *p* value are indicated. *Abbreviations: HRCT* high resolution computed tomography, *SpO*_*2*_ oxygen saturation by pulse oximetry, *6MWT* six minute walk test

**Fig. 2:**
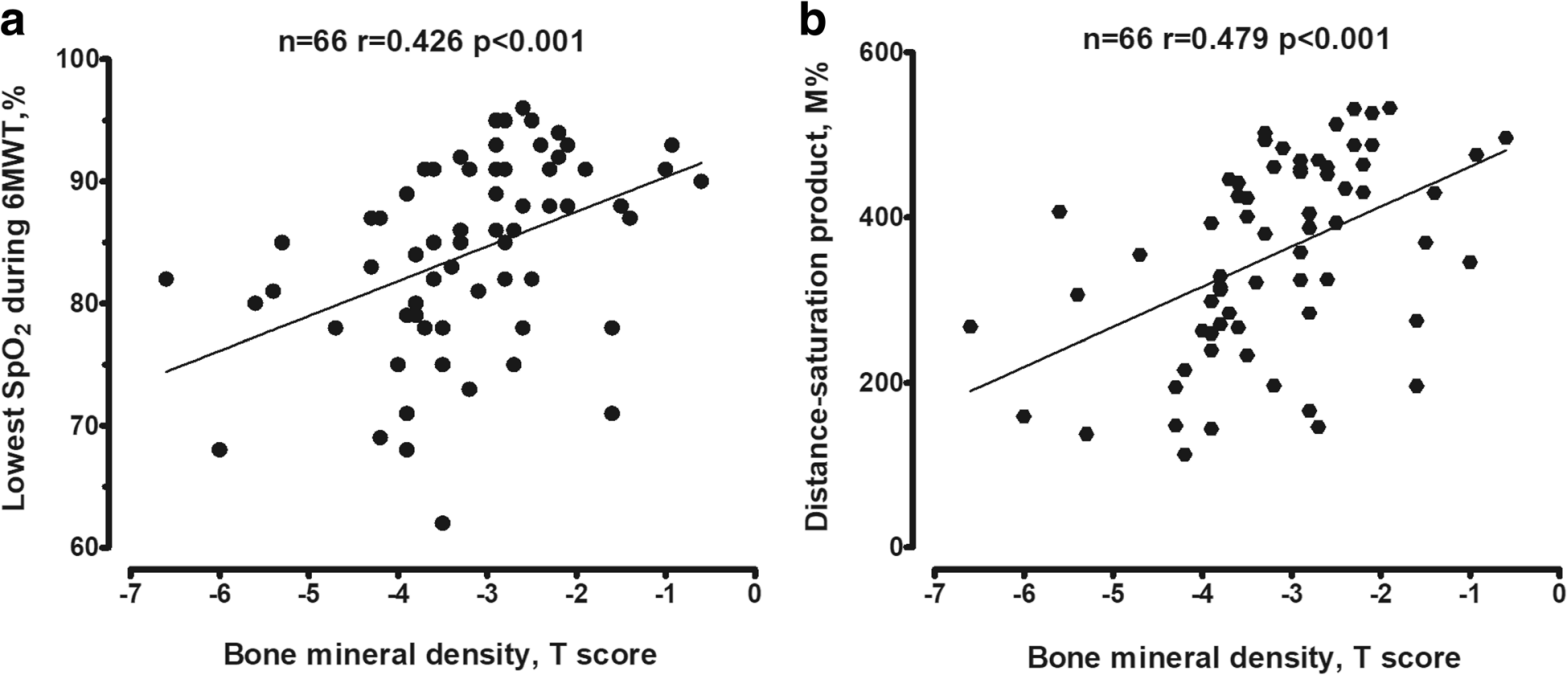
Correlation between bone mineral density (T score) and the lowest SpO_2_ during 6MWT (**a**) and distance-saturation product (**b**). *Abbreviations*: *SpO*_*2*_ oxygen saturation by pulse oximetry, *6MWT* six minute walk test

**Fig. 3:**
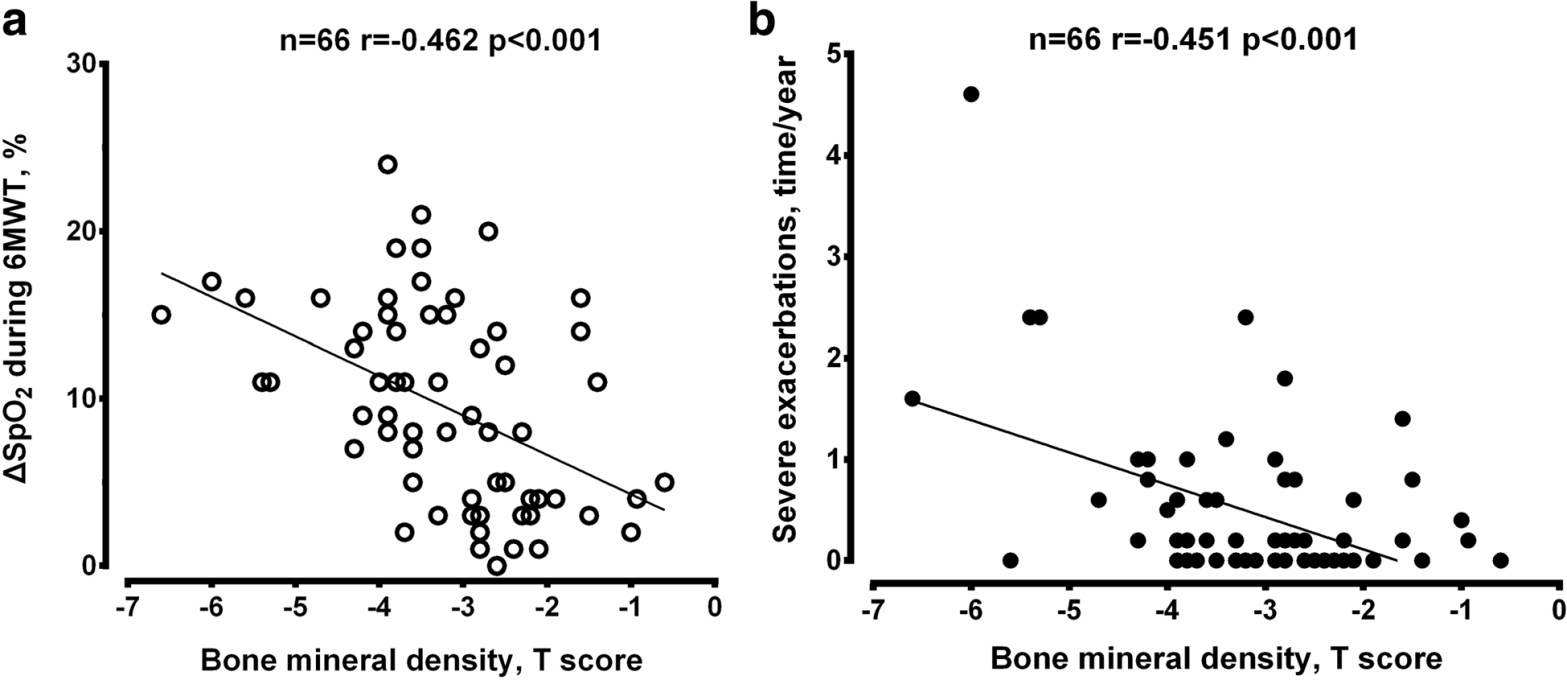
Correlation between bone mineral density (T score) and the ΔSpO_2_ during 6MWT (**a**), severe exacerbations (**b**). *Abbreviations*: *6MWT* six minute walk test, *ΔSpO*_*2*_ fall of oxygen saturation by pulse oximetry defined as 100 X (pre-6MWT-lowest-6MWT)/pre-6MWT

The nadir of exertional SpO_2_ during the 6MWT was significantly negatively correlated with HRCT score (*r* = − 0.538, *p* < 0.01), suggesting an association between the extent of lung destruction and oxygen desaturation (Fig. 1). BMD was correlated positively with the lowest SpO_2_ during the 6MWT (*r* = 0.426, *p* < 0.01) (Fig. 2a) and DSP (*r* = 0.479, *p* < 0.01) (Fig. 2b), but negatively with the fall of saturation (ΔSpO_2_) (*r* = − 0.462, *P* < 0.01) (Fig. 3a) and severe exacerbations (*r* = − 0.451, *p* < 0.01) (Fig. 3b). No significant correlation was noted between BMD and HRCT score.

In univariate analysis, BMI, desaturation during 6MWT, 6MWD, the lowest SpO_2_ during 6MWT, FEV_1_, severe exacerbation and short-term systemic corticosteroid loading were significantly associated with BMD at the level of femoral neck (Table 2), while age, gender and HRCT score were not. The full model of the multivariate analysis was present in Table 3, and the model R^2^ was 0.50. And then stepwise multivariate regression analysis (Table 3) was used to evaluate the relative contribution of each variable to predict the change in BMD and, as a result, age, BMI, being a desaturator, and severe exacerbation were the selected independent factors for BMD (with R^2^ of 0.48) finally due to colinear issue between the variables. In addition, short-term systemic corticosteroid loading was added to the model, but it was not found to be an independent factor because it correlated with severe exacerbation (Table 3).

**Table 2:** Univariate regression analysis of variables associated with BMD at femoral neck

Parameter	beta	Standard error	95% CI	t	*p*-value
Gender	0.435	0.341	−0.247~ 1.117	1.275	0.207
Age (year)	−0.024	0.014	−0.051 ~ 0.004	−1.698	0.094
BMI (kg/m^2^)	0.066	0.029	0.008 ~ 0.123	2.271	0.027*
Desaturation	−1.228	0.270	−1.768 ~ −0.687	−4.541	< 0.001*
HRCT score	−0.047	0.031	−0.108 ~ 0.014	−1.547	0.127
Lowest SpO_2_ during 6MWT (%)	0.064	0.017	0.03 ~ 0.098	3.770	< 0.001*
6MWD (M)	0.004	0.001	0.002 ~ 0.006	3.636	0.001*
FEV_1_(L)	0.756	0.253	0.25 ~ 1.262	2.986	0.004*
Severe exacerbation	−0.134	0.033	−0.201~0.068	−4.040	< 0.001*
Short-term systemic corticosteroid loading	−0.065	0.021	−0.106 ~ 0.024	−3.148	0.002*

*Abbreviations*: *HRCT* high resolution computed tomography, *BMI* body mass index, *6MWT* six minute walk test, *SpO*_*2*_ oxygen saturation by pulse oximetry, *6MWD* six minute walk distance, *FEV*_*1*_ forced expiratory volume in 1 s, *CI* confidence interval

**Table 3:** Stepwise multivariate regression analysis of variables associated with BMD at femoral neck

	Full model					Stepwise				
Parameter	beta	Standard error	95% CI.	t	*p*-value	beta	Standard error	95% CI	t	*p*-value
Gender	0.276	0.312	−0.351~ 0.903	0.884	0.381					
Age (yr)	−0.034	0.013	−0.061 ~ − 0.008	−2.603	0.012	− 0.036	0.011	− 0.057 ~ − 0.015	−3.396	0.001
BMI (kg/m^2^)	0.065	0.026	0.012 ~ 0.117	2.462	0.017	0.060	0.022	0.016 ~ 0.105	2.711	0.009
Desaturation	−0.937	0.337	−1.615 ~ − 0.260	−2.778	0.008	−0.913	0.228	−1.37 ~ − 0.456	−3.999	< 0.001
HRCT score	0.035	0.032	−0.030 ~ 0.100	1.078	0.286					
Lowest SpO_2_ during 6MWT (%)	0.003	0.023	−0.043 ~ 0.050	0.146	0.885					
6MWD (M)	0.001	0.001	−0.002 ~ 0.004	0.557	0.58					
FEV_1_(L)	−0.032	0.301	−0.636 ~ 0.572	− 0.107	0.916					
Severe exacerbation	−0.059	0.06	−0.180 ~ 0.062	− 0.975	0.334	−0.078	0.028	−0.134 ~ − 0.022	−2.807	0.007
Short-term systemic corticosteroid loading	−0.01	0.035	−0.081~ 0.060	− 0.291	0.772					

Abbreviations: *HRCT* high resolution computed tomography, *BMI* body mass index, *6MWT* six minute walk test, *SpO*_*2*_ oxygen saturation by pulse oximetry, *6MWD* six minute walk distance, *FEV*_*1*_ forced expiratory volume in 1 s, *CI* confidence interval

## Discussion

To the best of our knowledge, this is the first study to investigate the severity and risk factors for osteoporosis in Asian non-CF bronchiectasis patients. Of our patients with non-CF bronchiectasis, 68% exhibited exertional desaturation during the 6MWT and osteoporosis was evident in 82% desaturators and 43% non-desaturators. The lowest SpO_2_ during 6MWT and DSP were both positively correlated with BMD. In multivariate analysis, desaturation during the 6MWT was the factor most closely related to osteoporosis in patients with non-CF bronchiectasis. Other risk factors included old age, low BMI, and severe exacerbation. Thus, desaturation during 6MWT was highly associated with osteoporosis in patients with non-CF bronchiectasis.

Osteoporosis ranks amongst the top five comorbidities associated with non-CF bronchiectasis and 15.9% of a multicenter European non-CF bronchiectasis cohort (986 patients) had osteoporosis [3], while in a much smaller cohort of 20 of bronchiectasis patients, 25% had osteoporosis [4]. Our results, while supporting the findings of the previous studies [3, 4], further revealed that Asian populations with non-CF bronchiectasis appear to have more severe osteoporosis (70%) than non-Asian populations. Ethnic differences may also be involved, although the true incidence of Asian non-CF bronchiectasis-associated osteoporosis is unclear.

The detailed mechanism involved in the development of osteoporosis in patients with non-CF bronchiectasis is still unknown. In the multivariate analysis, the major factors affecting BMD were old age, desaturation during 6MWT, low BMI, and the number of severe exacerbation events. The possible pathogenesis of osteoporosis is multifactorial, and old age and low BMI have been known to contribute to low BMD [6]. The surprising link of the study is that we found a high degree of association between exertional desaturation and osteoporosis, which may provide new insight into the underlying pathogenesis of this disease. Hypoxia is an important pathophysiological change in non-CF bronchiectasis due to lung structural damage and airflow obstruction, and these patients are likely to experience desaturation during exercise. In this study, HRCT scores were correlated with the lowest SpO_2_ during the 6MWT. Notably, most of these patients had sufficient SpO_2_ at rest but developed severe desaturation during walking, which could constitute an intermittent hypoxia model. Intermittent hypoxia is associated with bone resorption and abnormal bone metabolism in patients with obstructive sleep apnea [9]. Osteogenic–angiogenic coupling is regulated by the HIF-1α transcription factor [17], which accumulates in response to hypoxia [15]. Thus, HIF-1α directly promotes osteoclast activities [16], directly inhibits osteoblast differentiation [17], and blocks anabolic actions of parathyroid hormone on bone formation in mature mouse [18]. HIF-1α induces the production of vascular endothelial growth factor (VEGF). VEGF stimulates osteogenic differentiation and the subsequent proliferation and survival of osteoblasts [17], and also stimulates hematopoietic stem cell differentiation into osteoclasts and increases bone resorption in humans [27]. Hypoxia affects bone metabolism and microarchitecture [28] and induces osteoporosis change by blocking the growth and differentiation of osteoblasts and strongly stimulates the formation of osteoclast [29, 30]. HIF-1α and VEGF both promote the activities of osteoclasts but have opposing effect on osteoblasts under different conditions, such as hypoxia. Taken together, hypoxia may affect bone cell function and can be considered as a risk factor for the development of osteoporosis [30], especially in non-CF bronchiectasis patients with desaturation during walking or exercise.

The vicious circle of bronchiectasis results from repetitive infection and systemic inflammation [31]. Acute exacerbations reflect the overall severity of inflammation and infection in clinical practice. The frequent attacks result in decreased lung function and increased mortality [32]. In our study, frequent severe exacerbation was also independently associated with osteoporosis. Previous studies have found that systemic inflammatory markers such as C reactive protein and airway cytokines (tumor necrosis factor-α, IL-1, IL-8) are increased in patients with bronchiectasis [32–34]. IL-1, IL-6 and TNF-α are strong inducers of bone resorption and subjects with highly expression of these cytokines are susceptible to develop osteoporosis [35, 36]. In addition, hypoxia may promote bacterial infection, enhance the activation of hypoxia inducible factors (HIF) and nuclear factor (NF)-κB, and propagate systemic inflammation or increase recurrent exacerbations [37, 38]. Pro-inflammatory cytokines disturb the balance of bone metabolism, and stimulate osteoclast function through the receptor activator of nuclear factor-B (RANK) and its functional ligand (RANKL), such as TRANCE (TNF-related activation-induced cytokines) and macrophage colony stimulating factor (M-CSF) [7]. Thus, the interaction of hypoxia and inflammation may aggravate osteoporosis in non-CF bronchiectasis patients who experience desaturation.

Bronchiectasis patients were treated with oral or parenteral corticosteroids during a hospitalised exacerbation [39]. Oral corticosteroid treatment is known to cause loss of bone density and the cumulative corticosteroid dose can be a major significant predictor for bone loss [40]. We have shown that the cumulative dose of systemic corticosteroids was higher in bronchiectasis patients who desaturated because of frequent exacerbation. In univariate analysis, the loading dose of systemic steroids was also one of risk factor for osteoporosis in non-CF bronchiectasis with desaturation. However, in multivariate analysis, the cumulative dose of systemic corticosteroids which was not an independent risk for osteoporosis in non-CF bronchiectasis was less important than desaturation and acute exacerbation. There is currently no control randomized study supporting the use of oral corticosteroids in non-CF bronchiectasis either for short-term (during an exacerbation) or long-term use [41]. In light of our observations, overuse systemic corticosteroid or cumulative steroid loading during acute exacerbations that may predispose to diminished bone density is warning. Nebulized or oral antibiotics combined with airway clearance instead of oral systemic steroids are mandatory for treatment of bronchiectasis exacerbation [39], thus attenuating the potential aggravation of osteoporosis in non-CF bronchiectasis.

The 6MWT is a convenient clinical evaluation tool and many variables derived from the 6MWT are useful for evaluating prognosis [42]. The current research results indicate that the lowest oxygen saturation value and DSP were correlated with BMD. DSP is a composite measure that reflects both exercise capacity and desaturation. Therefore, DSP could be used to evaluate physical activity, which is an important factor associated with osteoporosis. 6MWT may provide a useful tool to assess the clinical phenotype of non-CF bronchiectasis for clinicians, and identify the risk factors potentially influencing clinical care.

### Limitations

This study had some limitations. First, this study included the relatively small number of patients recruited from a single medical center, thus potentially limiting the generalizability of its findings. In analysis of desaturation contributing to osteoporosis between 21 non-desaturators and 45 desaturators, the enrolled number was adequate according to the power calculation. However, larger multicenter studies, particularly focused on the prevalence and risk of osteoporosis in Asian populations are necessary to corroborate our results. Second, we did not measure serum calcium, vitamin D, or parathyroid hormonal levels, which are plausible confounding factors. This may have led to bias. Third, it is well known that physical activity benefits bone metabolism and prevents the development of osteoporosis. We did not evaluate the level of physical activity between these two groups but the desaturators may have a lower level of physical activity. Thus, we cannot exclude the impact of physical activity that may contribute to diminished bone density in desaturators. Finally, we did not measure inflammatory cytokines and bone resorption marker levels; thus, we could not validate the association of osteoporosis with inflammation in non-CF bronchiectasis.

## Conclusion

Osteoporosis was a common comorbidity in non-CF bronchiectasis patients, particularly in those who were older, had low BMI, and exhibited frequent severe exacerbation. Desaturation during 6MWT was a strong independent predictive factor for osteoporosis. Future research is warranted to clarify the underlying pathogenesis. The 6MWT may be a useful tool to identify the clinical phenotype of non-CF bronchiectasis and thus raise clinician awareness regarding the disease, thus promoting early intervention, thereby preventing the development of osteoporosis or other comorbidities.

## Abbreviations

6MWD: 6 min walking distance
6MWT: 6 min walk test
BMD: Bone mineral density
CF: Cystic fibrosis
DSP: Distance–saturation product
DXA: Dual-energy X-ray absorptiometry
FEV_1_: Forced expiratory volume in one second
FVC: Forced vital capacity
HIF: Hypoxia-inducible factor
HRCT: High-resolution computed tomography
IL-1β: Interleukin-1β
IL-6: Interleukin-6
IL-8: Interleukin-8
M-CSF: Macrophage colony stimulating factor
NLRP3: Nucleotide-binding domain, leucine-rich-containing family, pyrin domain-containing-3 or Nod-like receptor protein 3
OSA: Obstructive sleep apnea
RANK: Receptor activator of nuclear factor-B
SpO_2_: oxygen saturation
TNF-α: Tumor necrosis factor-α
TRANCE: TNF-related activation-induced cytokines
VEGF: Vascular endothelial growth factor
ΔSpO_2_: fall in SpO_2_
